# Effect of a mixed reality-based intervention on arm, hand, and finger function on chronic stroke

**DOI:** 10.1186/s12984-016-0153-6

**Published:** 2016-05-11

**Authors:** Carolina Colomer, Roberto Llorens, Enrique Noé, Mariano Alcañiz

**Affiliations:** Servicio de Neurorrehabilitación y Daño Cerebral de los Hospitales NISA. Fundación Hospitales NISA, Valencia, Spain; Instituto Interuniversitario de Investigación en Bioingeniería y Tecnología Orientada al Ser Humano, Universitat Politècnica de València, Camino de Vera s/n, Valencia, 46022 Spain; Ciber, Fisiopatología Obesidad y Nutrición, CB06/03 Instituto de Salud Carlos III, Av. Sos Baynat s/n, Univesity of Jaume I, Castellón, 12071 Spain

**Keywords:** Stroke, Upper limb, Hemiparesis, Physical therapy, Virtual reality, Augmented reality, Tabletop systems

## Abstract

**Background:**

Virtual and mixed reality systems have been suggested to promote motor recovery after stroke. Basing on the existing evidence on motor learning, we have developed a portable and low-cost mixed reality tabletop system that transforms a conventional table in a virtual environment for upper limb rehabilitation. The system allows intensive and customized training of a wide range of arm, hand, and finger movements and enables interaction with tangible objects, while providing audiovisual feedback of the participants’ performance in gamified tasks. This study evaluates the clinical effectiveness and the acceptance of an experimental intervention with the system in chronic stroke survivors.

**Methods:**

Thirty individuals with stroke were included in a reversal (A-B-A) study. Phase A consisted of 30 sessions of conventional physical therapy. Phase B consisted of 30 training sessions with the experimental system. Both interventions involved flexion and extension of the elbow, wrist, and fingers, and grasping of different objects. Sessions were 45-min long and were administered three to five days a week. The body structures (Modified Ashworth Scale), functions (Motricity Index, Fugl-Meyer Assessment Scale), activities (Manual Function Test, Wolf Motor Function Test, Box and Blocks Test, Nine Hole Peg Test), and participation (Motor Activity Log) were assessed before and after each phase. Acceptance of the system was also assessed after phase B (System Usability Scale, Intrinsic Motivation Inventory).

**Results:**

Significant improvement was detected after the intervention with the system in the activity, both in arm function measured by the Wolf Motor Function Test (*p* < 0.01) and finger dexterity measured by the Box and Blocks Test (*p* < 0.01) and the Nine Hole Peg Test (*p* < 0.01); and participation (*p* < 0.01), which was maintained to the end of the study. The experimental system was reported as highly usable, enjoyable, and motivating.

**Conclusions:**

Our results support the clinical effectiveness of mixed reality interventions that satisfy the motor learning principles for upper limb rehabilitation in chronic stroke survivors. This characteristic, together with the low cost of the system, its portability, and its acceptance could promote the integration of these systems in the clinical practice as an alternative to more expensive systems, such as robotic instruments.

**Electronic supplementary material:**

The online version of this article (doi:10.1186/s12984-016-0153-6) contains supplementary material, which is available to authorized users.

## Background

Motor impairments are a common consequence of stroke and a major cause of disability [1]. Specifically, upper limb paresis is among the most significant deficits and represents an important obstacle for independence [2]. Impairment of upper limb motor function is present in more than 80 % of stroke survivors, and moderate dexterity after six months is only expected in 30 to 40 % of the cases [3].

It is commonly assumed that recovery of motor function after a brain injury involves neural reorganization of spared areas in both hemispheres to take over functions previously driven by the injured areas [4]. In fact, brain plasticity and behavior are interrelated: on one hand, behavior is a result of reorganized brain activity [1, 4]; on the other hand, adaptive neural reorganization is driven by skill-dependent experiences and behavior [4]. Nevertheless, reorganization is not driven by mere repetition. It only occurs when the experience implies learning [4]. Therefore, it can be deduced that motor rehabilitation should focus on driving plasticity by experiences that mean a challenge for the motor skills of the patients. In addition, motor learning principles, such as intensity, repetition, task-orientation, and feedback have proven to modulate the functional improvement after stroke [5–9].

Virtual Reality (VR) is an especially interesting research field since it allows to create computer-generated environments and provide customized experiences involving different sensory channels, commonly sight, hearing, and/or touch [10]. An increasing number of studies report promising results of its application to motor rehabilitation after stroke [10, 11], specifically for upper limb [11–13]. First, movement kinematics when reaching, grasping, transporting, and releasing objects in a virtual environment are comparable to those in the physical world, thus suggesting that the training of arm movements in VR can be a feasible alternative [14]. Second, VR has been shown effective at improving upper limb movements for reaching and grasping tasks involving proximal segments and global arm movements, in individuals with stroke in both acute and chronic stages [11, 13]. Third, distal fine motor control has also been effectively improved using VR, generally combined with robotic-like devices [2, 15, 16]. Fourth, controlled trials suggest that VR may be beneficial to improve upper limb function and performance in activities of daily living, to a greater extent than same dosage of conventional therapy [3]. Finally, mixed-reality systems involving virtual and tangible objects may be useful in improving both functionality and the kinematics of reaching [17, 18]. Mixed-reality systems are particularly interesting because they combine interesting features of VR with tangible objects that subjects must manipulate. For instance, proprioceptive feedback has been suggested to exploit multimodal aspects of the observation of goal-oriented movements and the feedback on one’s actions [12]. However, clinical research so far with these systems has mainly focused on shoulder and elbow training without specific involvement of hand and finger dexterity.

Basing on the existing evidence, we have developed a mixed reality system that satisfies the motor learning and neural plasticity principles to promote the rehabilitation of task-directed movements of the paretic upper limb involving hands and fingers. The system fits the motor condition of each subject allowing the training of a wide spectrum of movements, from gross proximal movements to finger dexterity, while being portable and inexpensive, in contrast to robotic systems. The objective of this paper is twofold: first, to determine the clinical effectiveness of an experimental intervention with the system to improve the motor function of arm, hand, and fingers in individuals with chronic stroke; and second, to determine the acceptance of this intervention as defined by users’ ratings of usability and motivation.

## Methods

### Subjects

All the outpatients who had suffered a stroke and presented a residual hemiparesis derived from the lesion, and were attending a long-term rehabilitation program in the Brain Injury Service of NISA Hospitals were potential candidates to participate in the study. Inclusion criteria were 1) age ≥ 35 and < 65 years old; 2) chronicity > 6 months; 3) no increase or slightly increase in muscle tone as defined by Modified Ashworth Scale [19] < 3; 4) ability to move the joints (proximal and distal) as defined by Medical Research Council Scale for Muscle [20] ≥ 2; 5) fairly good motor condition as defined by Motricity Index [21] ≥ 55; 6) absence of severe cognitive impairment as defined by Mini-Mental State Examination [22] > 23; and 7) able to follow instructions as defined by Mississippi Aphasia Screening Test [23] ≥ 45. The exclusion criteria were 1) individuals with ataxia or any other cerebellar symptom; 2) orthopedic alterations or pain syndrome of the upper limb; 3) peripheral nerve damage affecting the upper extremities; 4) individuals whose visual or hearing impairment does not allow possibility of interaction with the system; and 5) individuals with severe hemispatial neglect. Ethical approval for the study was granted by the Institutional Review Board of NISA Hospitals. All the eligible candidates who agreed to take part in the study were required to provide informed consent.

### Materials

#### Hardware setting

**Fig. 1:**
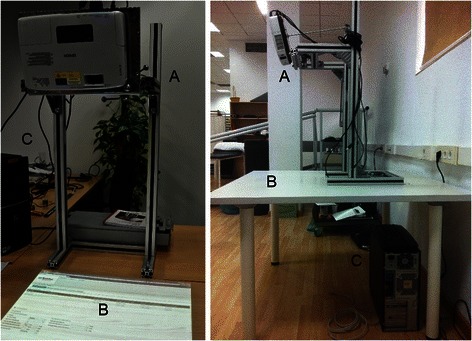
Prototype of the virtual reality-based system. The hardware used in this experiment consisted of: **a**) a Kinect™ (Microsoft®, Redmond, WA, USA), which estimated the depth information of the scene, and a LCD projector EB-1720 (EPSON, Suwa, NGN, Japan), which projected the VR; **b**) a conventional table; and **c**) a computer Vostro 420 (Dell Inc., Round Rock, TX, USA) equipped with a QuadCore @ 2.83 GHz and 4 GB of RAM, which generated the VE, tracked the movements of the user on the area of interest, and modified the VE according to them

**Fig. 2:**
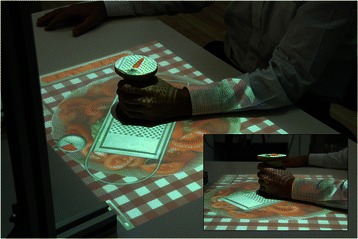
Participant training with the system. A participant interacts with the system using a tangible object. Participant must grate a carrot, represented on the top surface of the item, on a salad. The task is achieved through repeated flexion-extension of the wrist, while maintaining the forearm on the table and the elbow still

The mixed reality rehabilitation system consisted of a projective tabletop system that allowed multitouch interaction with the hands or via manipulation of tangible objects (Fig. 1). Essentially, the system consisted of a Kinect™ depth sensor (Microsoft®, Redmond, WA, USA) and a projector EB-1720 (Epson®, Suwa, Japan) separated 8 cm and attached to the upper plane of a rigid frame at 70 cm of height. The system was 95 x 70 x 40 cm and was fully portable. The sensor and the projector pointed down so that when the frame was placed on a table their field of view overlapped on its surface, thus defining an area of interaction of 55 x 40 cm^2^ [24]. The system projects a virtual environment on that area, which reacts according to the users’ movements, mimicking the interaction with the real world. In each exercise, the required movements of the upper limb segments, fingers, and tangible objects were detected from the depth information of the scene, tracked, and the interaction with the virtual objects was calculated to update the virtual environment (Fig. 2) (See Additional file 1 for more information).

#### Exercises

**Fig. 3:**
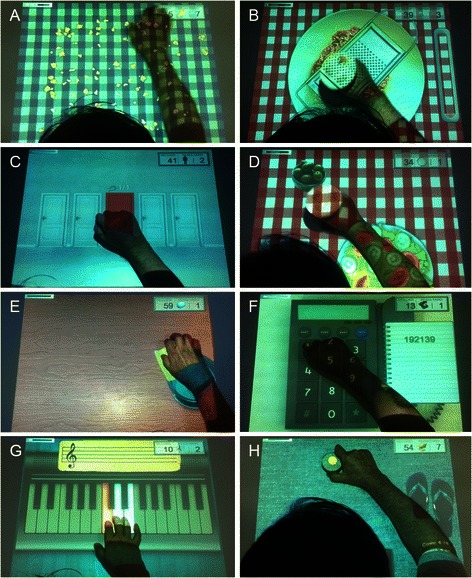
Description of the exercises. The exercises covered a wide range of hand and arm movements, mostly focusing on the flexion and extension of the elbow and the wrist. **a** *Exercise*: to sweep the crumbs from the table. *Movement*: flexion-extension of the wrist without involving the fingers. **b** *Exercise*: to grate. *Movement*: Grasping and flexion-extension of the wrist. **c** *Exercise*: to knock on doors. *Movement*: flexion-extension of the wrist against gravity. **d** *Exercise*: to cook. *Movement*: grasping involving flexion-extension of the elbow and rotation of the shoulders. **e** *Exercise*: to squeeze a sponge. *Movement*: flexion-extension of the metacarpophalangeal-interphalangeal joint. **f** *Exercise*: to dial a number. *Movement*: tapping. **g** *Exercise*: to play piano. *Movement*: flexion-extension of the thumb, index, and middle finger. **h** *Exercise*: to buy items. *Movement*: pincer grasping with the thumb and index involving flexion-extension of the elbow and rotation of the shoulders

The exercises consisted of a wide range of planar unimanual tasks that involved arm and hand movements, focused on the flexion and extension of the elbow, the wrist, and the metacarpophalangeal joint, and represented tasks that were likely to belong to the participant’s motor repertory (previous to the onset), aiming to maximize the relationship with activities of daily living (Fig. 3). The interaction with some exercises required tangible objects of different sizes to be grasped and moved. Handles with different thickness were available. Within each exercise, participants had to perform a task (to grate an item, to dial a number, etc.) as many times as possible. The task, in turn, was achieved if a number of repetitions were performed accurately enough within a time interval. The system controlled compensation during the exercises, requiring those segments not involved in the movement to be fixed in certain position. For instance, in the grating exercise, the forearm had to remain still and on the table while flexing and extending the wrist. Otherwise, repetitions were not valid (See Additional file 1 for more information). The difficulty of the exercises was determined by adjusting the required speed, number of repetitions, and accuracy of the movements. Before the intervention, therapists defined different levels of difficulty for each exercise by varying these parameters. After each exercise, the success rate was estimated as the percentage of tasks successfully achieved. When the success rate was higher than 80 %, the system automatically increased the level of difficulty. When the success rate was lower than 20 %, the system decreased the level. Exercises provided audiovisual feedback of the virtual environment and showed information about the remaining time, the repetitions successfully completed, and the previous records achieved by the participant. During the exercises, positive audiovisual reinforcement was provided when a task was achieved. In case of the task was not achieved, a negative feedback was provided. After each exercise, the system provided the success rate achieved.

### Procedure

A reversal (A-B-A) design was chosen to characterize the effects of the experimental intervention and to quantify the maintenance of gains. Phase A consisted of 30 training sessions of conventional physical therapy, and phase B consisted of 30 sessions of an experimental intervention with the mixed reality system. This design allowed to determine the effects of physical therapy, the effects of the experimental intervention, and the maintenance of gains after it when returning to physical therapy. The duration of both interventions was paired. In both phases, sessions were 45-min long and were administered three to five days a week. All the training sessions were supervised by a physical therapist, who in case of compensation, provided a tactile cue to correct the performance. No concomitant therapies were administered.

The conventional physical therapy intervention included active upper extremity tasks equivalent to those trained by the mixed reality system, which involved shoulder, elbow, wrist, and fingers and grasping of different items (in the absence of a virtual feedback). For example, the exercise that simulated knocking on doors in the mixed reality system (Fig. 3c) was matched with repetition of knocking movements (flexion-extension of the wrist with the forearm still) on square-shaped pieces of paper placed on a table. Two two-minute breaks were allowed after 15 and 30 min of the beginning of the session. The difficulty of the training was determined by a physical therapist in a previous exploratory session. During the intervention, exercises gradually increased in resistance (weights) and in repetitions. The experimental intervention included eight exercises in randomized order (Fig. 3). Duration of the exercises was set to five minutes each. Two-minute breaks were allowed after the third and sixth exercise. The difficulty of the experimental intervention was also initially determined in a previous exploratory session, and was automatically adjusted by the mixed reality system during the intervention or by the physical therapist who supervised the sessions to correct one-time alterations related to pain, motor performance, or inattention. The thickness of the handles of the tangible objects was also determined in the exploratory session to fit the grasp opening of each subject.

All the participants were assessed by a physical therapist, who was blind to the design of the study, 1) at the beginning of the initial phase A (A_i_); 2) at the end of the initial phase A, which was the beginning of phase B (B_i_); at the end of phase B, which was the beginning of the second phase A (B_f_), at the end of the second phase A (A_f_). In accordance with the International Classification of Functioning, Disability and Health [25], the assessment protocol evaluated 1) the body structures, with the Modified Ashworth Scale [26]; 2) the body functions, with a strength test with a dynamometer [27], the Motricity Index, and the upper extremity subscale of the Fugl-Meyer Assessment Scale [28]; 3) the body activities, with the Manual Function Test [29], the Wolf Motor Function Test [30], the Box and Blocks Test [31], and the Nine Hole Peg Test [32]; and 4) the participation, with the subscales of Quality of Movement and Amount of Use of the Motor Activity Log [33]. In addition, acceptance of the experimental system was assessed in B_f_ with the System Usability Scale [34] and with four subscales of the Intrinsic Motivation Inventory [35]. The System Usability Scale is a simple ten-item scale that serves as a global assessment of subjective usability. It employs a Likert scale with scores ranging from 0 to 100. The Intrinsic Motivation Inventory is a multidimensional questionnaire structured into various subscales. Each subscale includes different questions rated on a seven-point Likert scale. In this study, this questionnaire was used to assess participant interest/enjoyment, perceived competence, pressure/tension, and value/usefulness measures. Scores approaching seven in each subscale represent positive values in terms of motivation, with the exception of the pressure/tension subscale, for which high scores represent high levels of tension.

### Statistical analysis

For each scale and test, scores in all the assessments were compared using repeated measures analyses of variance (ANOVA). ANOVA findings that violated the sphericity assumption were accommodated by Greenhouse and Geisser’s conservative degrees of freedom adjustment. Post-hoc simple contrasts (Bonferroni) were conducted for each significant time main effect to determine the source of the significant difference. Data were confirmed to have a normal distribution using the Shapiro–Wilks normality test. The α level was set at 0.05 for all analyses (two-sided). All analyses were computed with SPSS for Mac, version 15 (SPSS Inc., Chicago, USA).

## Results

### Subjects

A cohort of 108 individuals with stroke were examined for eligibility. A sample of 32 participants (29.6 %) satisfied the inclusion criteria in the study and accepted to participate. All of them were enrolled. Two subjects were discharged and dropped out the study, consequently, their data were not included for analysis. The final sample (17 men and 13 women) was aged 58.3 ± 10.1 years old and had a chronicity of 357.5 ± 270.1 days. Lesions were ischemic (*n* = 17) or hemorrhagic (*n* = 13), with a preponderance of right-sided occurrence (*n* = 17). Ischemic lesions presented total anterior circulation infarcts (*n* = 4), partial anterior circulation infarcts (*n* = 9), and lacunar circulation infarcts (*n* = 4).

### Clinical effectiveness

**Fig. 4:**
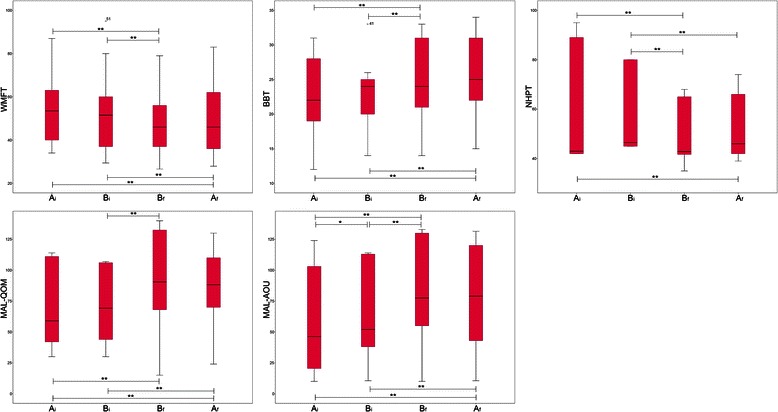
Statistically significant effects throughout the intervention. Significant improvement was detected after the experimental intervention (from B_i_ to B_f_) but not after the following conventional intervention (from B_f_ to A_f_) nor the previous (from A_i_ to B_i_), but in the Amount of Use subscale of the Motor Activity Log. WMFT: Wolf Motor Function Test; BBT: Box and Blocks Test; NHPT: Nine Hole Peg Test; MAL-QOM: Quality of Movement subscale of the Motor Activity Log; MAL-AOU: Amount of Use subscale of the Motor Activity Log. *: *p* < 0.05; **: *p* < 0.01

Repeated measures ANOVA at every assessment of the clinical trial revealed a significant time effect in most of the scales that assessed the body activities (the Wolf Motor Function Test, the Box and Blocks Test, and the Nine Hole Peg Test) and in the participation, and a strong trend towards significance in the Fugl-Meyer Assessment Scale (Table 1). With respect to these scales throughout the therapy, post-hoc analysis showed significant improvement after the experimental intervention (from B_i_ to B_f_). However, this improvement was detected neither after the following conventional intervention (from B_f_ to A_f_) nor the previous (from A_i_ to B_i_), but in the Amount of Use subscale of the Motor Activity Log (Fig. 4). No significant differences were detected in either the body structures or functions.

**Table 1:** Clinical data

Measure	Start of phase A	Start of phase B	End of phase B	End of phase A	Significance
	(A_i_)	(B_i_)	(B_f_)	(A_f_)	
Modified Ashworth Scale					
Proximal	0.5 ± 0.5	0.5 ± 0.5	0.4 ± 0.5	0.3 ± 0.5	NS (*p* = 0.090)
Distal	0.3 ± 0.5	0.3 ± 0.5	0.3 ± 0.4	0.3 ± 0.4	NS (*p* = 0.400)
Dynamometer (kg)	32.2 ± 14.3	32.0 ± 12.8	33.2 ± 12.7	31.7 ± 12.1	NS (*p* = 0.240)
Motricity Index	73.2 ± 11.9	72.1 ± 12.5	73.3 ± 12.9	73.6 ± 12.12	NS (*p* = 0.100)
Fugl-Meyer Assessment Scale. Upper extremity subscale	50.2 ± 5.0	50.2 ± 5.0	51.1 ± 4.8	50.9 ± 4.9	NS (*p* = 0.061)
Manual Function Test	19.3 ± 3.6	18.9 ± 3.6	19.2 ± 3.5	19.3 ± 3.6	NS (*p* = 0.090)
Wolf Motor Function Test (s)	53.9 ± 15.7	52.2 ± 16.6	48.1 ± 15.7	49.5 ± 16.1	Bf < Bi** (*p* = 0.001)
					Bf < Ai** (*p* < 0.001)
					Af < Bi* (*p* = 0.010)
					Af > Ai** (*p* < 0.001)
Box and Blocks Test (blocks)	22.4 ± 5.2	22.3 ± 4.4	24.8 ± 5.4	25.3 ± 5.3	Bf > Bi** (*p* = 0.001)
					Bf > Ai** (*p* < 0.001)
					Af > Bi** (*p* < 0.001)
					Af > Ai** (*p* < 0.001)
Nine Hole Peg Test (s)	63.1 ± 4.3	60.4 ± 3.2	50.9 ± 2.2	52.5 ± 2.3	Bf < Bi** (*p* < 0.001)
					Bf < Ai** (*p* < 0.001)
					Af < Bi** (*p* < 0.001)
					Af > Ai** (*p* < 0.001)
Motor Activity Log – Quality of Movement	68.5 ± 30.2	70.4 ± 26.3	88.5 ± 38.9	84.2 ± 32.1	Bf > Bi** (*p* < 0.001)
					Bf > Ai** (*p* < 0.001)
					Af > Bi** (*p* < 0.001)
					Af < Ai** (*p* < 0.001)
Motor Activity Log – Amount of use	56.3 ± 38.2	61.6 ± 34.9	79.4 ± 39.7	75.9 ± 40.7	Bi > Ai* (*p* = 0.015)
					Bf > Bi** (*p* < 0.001)
					Bf > Ai** (*p* < 0.001)
					Af > Bi** (*p* < 0.001)
					Af < Ai** (*p* < 0.001)

Results are expressed in terms of mean and standard deviation. In case of significance was detected in each scale, only significant temporal relationships are shown. NS: no significant; * *p* < 0.05; ** *p* < 0.01

### Acceptance

With regards to the usability, scores in the System Usability Scale (79.13 ± 7.54 from a total score of 100) showed good acceptance of the experimental system. According to the Intrinsic Motivation Inventory, participants reported high levels of interest and enjoyment (5.73 ± 0.79 of 7), found themselves competent (5.21 ± 0.98) but not pressured (1.98 ± 0.58), and considered the intervention useful (6.17 ± 0.69).

## Discussion

This study evaluates the effectiveness and acceptance of a low-cost mixed reality instrument that provides intensive task-oriented exercises for arm, hand, and finger function rehabilitation in a population of chronic stroke survivors with hemiparesis. Positive effects of the experimental intervention were detected in both activity and participation, and also influenced the progression of the participants.

The significant improvement in timed tests related to activity after the experimental intervention must be highlighted, since task performance is considered an indicative of functional improvement in individuals with chronic stroke [36], and since movement speed and quality of movement are interrelated [37]. Our results supports previous findings using mixed reality systems in the Wolf Motor Function Test [17]. Interestingly, changes detected by the Wolf Motor Function Test have been reported to be of clinical importance [37]. The strong tendency towards statistical significance detected in the Fugl-Meyer Assessment Scale are also in line with previous reports [17, 18]. The different nature of this scale and the Wolf Motor Function Test and the chronicity of our sample could have prevented greater effects. This scale has been shown to be more sensitive in the acute phase [38] and for chronicity of less than six months [39]. However, it may separate motor recovery from functional recovery and, therefore, may not be responsive to functional improvements in chronic populations [40]. The Fugl-Meyer Assessment Scale focuses on multijoint upper extremity function and examines synergy patterns that may no longer form the basis of our intervention [41]. Moreover, it is a 3-point scale and do not differentiate changes in the less affected extremity. In contrast, the Wolf Motor Function Test assesses the performance time involving single joint or interjoint movements, which were frequently engaged in our intervention. The significant improvement in the gross manual dexterity, assessed by the Box and Block Test, could have been facilitated by an improvement in control of the elbow and wrist synergies and the grasping mechanism promoted by the interaction with tangible objects, which supports previous findings [18]. In addition, the specific training of the flexion and extension of the wrist in different positions and the metacarpophalangeal and interphalangeal joint promoted by our system, could also explain the improvement detected in the Nine Hole Peg Test. It is important to highlight that previous research on stroke survivors involving some robotic systems has shown no improvement after intervention in the Box and Block Test [12, 42] unless the wrist joint [43] or finger dexterity [44] are specifically trained. However, these two last robotic systems failed to provide improvement reflected in the Nine Hole Peg Test, even in acute phase [45]. This should highlight the benefits of our system, since it can promote hand dexterity, as measured by the Box and Block Test and the Nine Hole Peg Test, while being cheaper and more portable than robotic systems.

Although clinical scales do not allow the ultimate distinction between true recovery and behavioural compensation [46, 47], the results suggest effective motor learning and motor skill retention derived from the experimental treatment. We hypothesize that the improvement in the clinical condition of the participants could be explained by the nature of the exercises, which satisfied the motor learning and neural plasticity principles. First, exercises were intensive and repetitive, characteristics that have been reported to influence improvement [5]. Second, they represented meaningful tasks specially designed to address functional activities, which has been reported of major importance for motor rehabilitation [5, 6] and is known to positively affect arm-hand function recovery and motor control in stroke patients [46]. Third, augmented extrinsic feedback, a major aspect of motor learning [6, 8, 46], was provided during the training in the visual, auditory, and tactile channels. Interestingly, auditory augmentation of visual feedback can be beneficial during the execution of upper limb movements [48]. Fourth, the training drove subject´s attention to the effect of the action, which has been reported to enhance learning [49]. Finally, the difficulty of the training was particularized to each participant in each session, which is essential for motor learning and neural reorganization [6, 46, 49]. Previous research has found that functional improvement, which has been associated with cortical reorganization by different neuroimaging studies [10, 50], can occur at any time [12, 51, 52]. However, the chronicity of the sample, which ensured that the functional improvement was externally driven by the intervention [1, 5], could have limited greater improvement. It is important to highlight though that clinical improvement provided by the experimental intervention was retained after the second A-phase, it is, after returning to physical therapy. The practice under varied conditions promoted by the experimental system could have supported this retention, which has been reported as a better indicator of motor learning than the performance during or just after the practice [6].

The limited results obtained in the body structure and in the body function domains may be related to task-specific effects of motor learning [5, 46]. In line with the tendency of the last decade to shift the efforts of hand-arm rehabilitation from the function level towards the activity and participation level [46], the mixed reality system was designed to train specific tasks that imply the use of the affected arm, hand, and fingers, without explicit focus on strength or joint movement. This orientation, together with the discrete nature of the Manual Function Test (with scores ranging from 1 to 4), and, again, with the chronicity of the sample, could have prevented significant improvement in these components.

The positive reports on perception of improvement and on the use of the paretic arm after the experimental intervention evidenced by the Motor Activity Log, and the high scores about usefulness and enjoyment evidenced by the Intrinsic Motivation Inventory, could depict a relationship between acceptance of the intervention and its repercussion to daily life. This fact could be explained by the ability of the system to motivate patients, which would support previous studies [12, 15, 51, 52]. Importantly, motivation is believed critical for learning [7, 49], and is considered one of the basic principles that should be satisfied by any rehabilitation approach [6, 9]. Finding the rehabilitation enjoyable is thought to increase the level of engagement, participation, and compliance [15], thus increasing the effectiveness of a rehabilitation program.

These results must be interpreted taking into account the limitations of the study. First, the characteristics of the sample are inherently linked to the specialized neurorehabilitation service where the study took place, which could restrict the generalization of the results. Second, no kinematic analysis was performed. Consequently, although compensatory strategies were restricted during the intervention, they were not controlled during the assessment, which could have influenced the performance in the scales and tests. Third, although the physical therapist who assessed the participants’ condition did not know the protocol, the therapists who administered and controlled the intervention were not blind. Fourth, the requirements of the system could restrict interaction of some individuals. Participants were required to have enough motor control to actively move the hemiparetic arm, hand, and fingers along the table and enough cognitive and communication skills to understand and follow instructions. Finally, the sample of the study (*n* = 30) actually can be considered as a small sample, which can also limit the extrapolation of the results.

However, the improvement detected in our sample supports the clinical effectiveness of mixed reality interventions that satisfy the motor learning and neural reorganization principles to improve upper extremity motor ability and finger dexterity in chronic stroke survivors. The effectiveness of the system together with its low cost, its portability, and its acceptance could promote its integration in the clinical practice as an alternative to more expensive systems, such as robotic instruments.

## Conclusions

The mixed reality intervention was shown to be effective and motivating for rehabilitation of the upper extremity motor ability and manual dexterity in chronic individuals with stroke. The low cost of the system, its portability, and its acceptance could promote its integration in the clinical practice as an alternative to more expensive systems.

## Additional file

Additional file 1: Operation of the tracking system. (PDF 175 kb)

## Abbreviations

VR: virtual reality
ANOVA: analyses of variance
