# Erratum to: Effect of a mixed reality-based intervention on arm, hand, and finger function on chronic stroke

**DOI:** 10.1186/s12984-017-0224-3

**Published:** 2017-02-14

**Authors:** Carolina Colomer, Roberto Llorens, Enrique Noé, Mariano Alcañiz

**Affiliations:** 1 Servicio de Neurorehabilitación y Daño Cerebral de los Hospitales NISA. Fundación Hospitales NISA, Valencia, Spain; 2 Universidad de Autónoma Barcelona, Barcelona, Spain; 3 Instituto Interuniversitario de Investigación en Bioingeniería y Tecnología Orientada al Ser Humano, Universitat Politècnica de València, Camino de Vera s/n, Valencia, 46022 Spain; 4 Ciber, Fisiopatología Obesidad y Nutrición, CB06/03 Instituto de Salud Carlos III, Av. Sos Baynat s/n, University of Jaume I, Castellón, 12071 Spain

## Erratum

The original article [1] mistakenly omitted a key affiliation for the author, Carolina Colomer. The authors would therefore like to state the affiliation of ‘Universidad Autónoma de Barcelona, Barcelona, Spain’ as the second affiliation for Dr Colomer.

Furthermore, the authors would also like to add a statement to the Acknowledgements sub-section stating:

“This work has been developed within the framework of a medical doctorate at the Universidad Autónoma de Barcelona.”
