# Supplementary material for: Effect of a mixed reality-based intervention on arm, hand, and finger function on chronic stroke
Source: J Neuroeng Rehabil. 2016 May 11;13:45. doi: 10.1186/s12984-016-0153-6 (PMC4864937; doi:10.1186/s12984-016-0153-6)
Supplement: Additional file 1: — Operation of the tracking system. (PDF 175 kb) [file 12984_2016_153_MOESM1_ESM.pdf]

Supplementary material

Operation of the tracking system

As stated in the main document, in each exercise, the required movements of the upper limb segments, fingers, and tangible objects are detected from the depth information of the scene and tracked. The interaction with the virtual objects is calculated from this information to update the virtual environment. Depth information is retrieved from the Kinect™ at a maximum frequency of 30 frames per second as a stream of grayscale images with a resolution at 640x480 pixels, where higher intensity represent less distance to the camera. The provision of the depth information as images facilitates the signal processing of these data through common image processing algorithms.

All of the exercises require a common processing that involves height delimitation and thresholding, which defines the binary large objects (blobs) in the image (Figure 1).

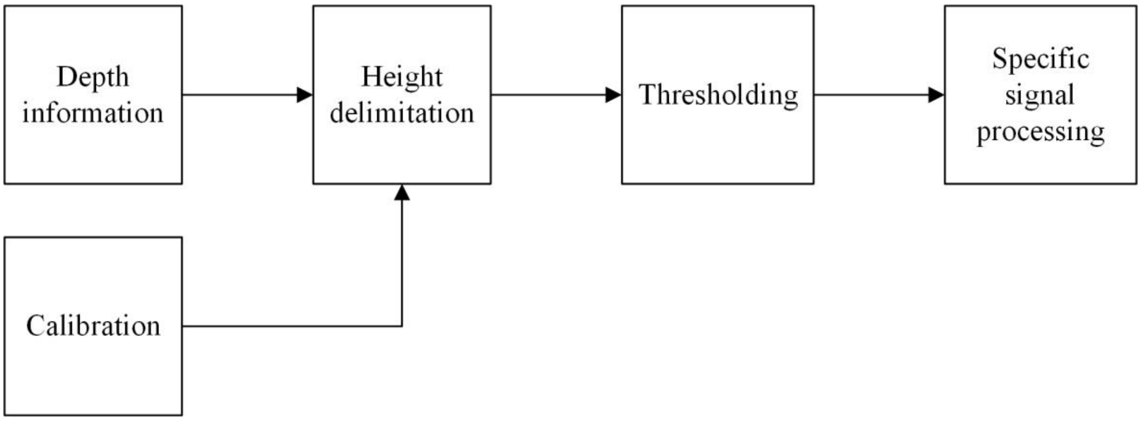

Figure 1. Diagram of the signal processing

The height delimitation process discards any information beyond the height detected in the calibration process, which is different for each subject and exercise. The thresholding process replaces each pixel in the depth image with a white pixel if the

intensity is greater (closer to the camera) than the intensity of those pixels that belong to the table plane and replaces the intensity of the other pixels with black. In addition, some exercises require specific processing to enable interaction with the virtual environment (Table 1).

Table 1. Signal processing and interaction

Exercise	Calibration	Processing	Interaction	Restrictions
To sweep the crumbs	Table plane, height of hand and forearm in semi pronation and in contact with the table	1. Height delimitation<br>2. Thresholding<br>3. Blob estimation	To sweep: Collision of the border of the hand and forearm with the crumbs	Forearm in contact with the table
To grate	Table plane, height of the tangible object[a]	1. Height delimitation<br>2. Thresholding<br>3. Blob estimation of forearm and upper surface of the tangible object<br>4. Eccentricity and size check of the surface[b]<br>5. Centroid estimation of the surface	To grate: Collision of the centroid with the center of the grater	Forearm still and in contact with the table
To knock on doors	Table plane, height of the hand and forearm in pronation with the palm down	1. Height delimitation<br>2. Thresholding<br>3. Blob estimation of forearm and fist	To knock: Collision of the fist with the door after a rise[d]	Forearm still and in contact with the table
To cook	Table plane, height of the tangible object[a]	1. Height delimitation<br>2. Thresholding<br>3. Blob estimation of upper surface of the tangible object	To pick up: Collision of the centroid with the ingredient<br>To place: Collision of the centroid with the plate	-

		4. Eccentricity and size check[b]<br>5. Centroid estimation		
To squeeze a sponge	Table plane, height and area of the hand with fingers extended	1. Height delimitation<br>2. Thresholding<br>3. Blob estimation<br>4. Estimation of the area and centroid of the hand	To absorb a water drop: Collision of the centroid of the hand with the water drop<br>To squeeze: Reduction of the area and raise of the hand while the centroid of the hand collides with the bucket[e]	-
To dial a number	Table plane, height of the hand with the index extended	1. Height delimitation<br>2. Thresholding<br>3. Blob estimation<br>4. Estimation of the fingertip[c]	To press: Collision of the fingertip with the button after a rise[d]	-
To play piano	Table plane, height of the hand with fingers extended	1. Height delimitation<br>2. Thresholding<br>3. Blob estimation<br>4. Estimation of the fingertips[c]	To press: Collision of the fingertip with the key after a rise[d]	Forearm in contact with the table
To buy items	Table plane, height of the tangible object[a]	1. Height delimitation<br>2. Thresholding<br>3. Blob estimation of upper surface of the tangible object	To pick up a coin: Collision of the centroid with the coin<br>To place: Collision of the centroid with the wallet	-

	4. Eccentricity and size check[b]<br>5. Centroid estimation	

The figure describes the workflow of the tracking system and the interaction within the virtual environment. [a]: A tangible object with a circular upper surface is required to interact with these exercises. The system, however, enables different handles to be used thus allowing to adjust the opening of the grasping. [b]: Eccentricity and size of the blobs are checked to discard false positives. The blob that represents the upper surface is required to have an eccentricity lower than 0.35. The threshold for the size is determined experimentally during the manufacture of the system. [c]: Estimation of the fingertips is performed using the convex hull algorithm [1]. [d]: Some exercises require the user to rise the fingers or the fist to interact. Only rises greater than a threshold are valid. Threshold can be adjusted for each subject and exercise. Only values greater than 1 cm are allowed to avoid false positives [2]. [e]: Interaction is only detected if the fingers are flexed and the hand is raised.

Feasibility of the tracking system

Even though the accuracy and resolution of Kinect™ depth data has been previously reported [2], a study was conducted to examine the feasibility of the system to track the required movements. The objective of the study was twofold: first, to determine the accuracy and the jitter of the estimated position of tangible objects and fingertips on the table plane; and second, to determine the accuracy and the jitter of the height estimation of fingertips on the table plane (0 cm) and 1 cm above it.

A 10 x 6 grid with 5 cm x 5 cm squares was defined on the table plane, covering an area of 50 x 30 cm^2. The center of two tangible objects (Figure 2) and the index fingertip of a right male mannequin hand were placed in all of the intersection points of the grid on the table plane. The index fingertip was also placed 1 cm above the table using a piece of wood. The position of the centroid and the fingertip at each point was estimated by the tracking system and registered during 5 s. The main accuracy and the jitter in the three spatial coordinates were calculated [3].

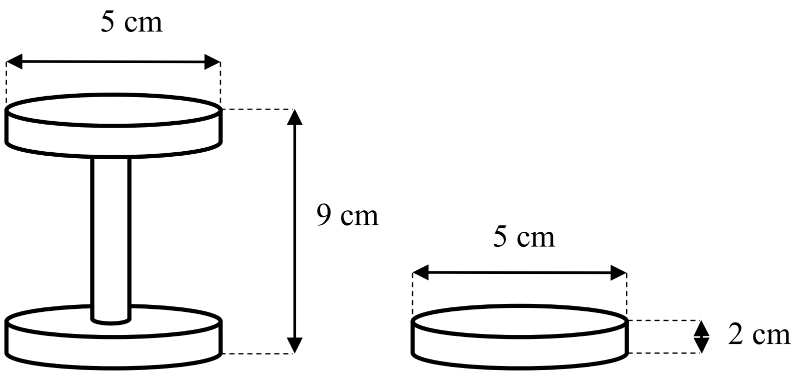

Figure 2. Tangible objects used in the study

With regards to the position on the table plane, the accuracy of the tracking system proved to be lower (better) for the tangible objects (2.3±0.3 mm for the 9-cm object, and 2.9±0.9 mm for the 2-cm object) than for the index fingertip (3.9±1.0 mm). The jitter

was similar for both objects (4.7±2.4 mm and 4.9±3.1 mm for the 9-cm and the 2-cm object, respectively) and also higher (worse) for the fingertip (5.8±3.3 mm). With regards to the height estimation of the fingertips, the tracking system had a mean error of 1.6±3.0 mm.

In general, worse results were achieved in the estimation of the position of the fingertip. The performance on the table plane could show a height dependency of the accuracy and/or an additional error caused by the convex hull algorithm. The tracking system also showed a relative error of 16% in the height estimation of fingertips, supporting previous reports on the performance of the Kinect™ [2]. However, the accuracy and jitter values for tangible objects and fingertips were not considered relevant for the proper performance of the system for two main reasons. First, values could be considered insignificant compared to the characteristics of the movement kinematics after stroke [4]. Second, the areas of interaction in the virtual environment were much larger than the maximum values of both parameters.

In consequence, the tracking system proved to be a feasible solution to facilitate the required interaction with the virtual environment through tangible objects and fingertips.

References

1. Graham, R.L., *An Efficient Algorithm for Determining the Convex Hull of a Finite Planar Set.* Information Processing Letters, 1972. **1**(4): p. 132-133.
2. Khoshelham, K. and S.O. Elberink, *Accuracy and resolution of Kinect depth data for indoor mapping applications.* Sensors (Basel), 2012. **12**(2): p. 1437-54.
3. Llorens, R., et al., *Tracking systems for virtual rehabilitation: objective performance vs. subjective experience. A practical scenario.* Sensors (Basel), 2015. **15**(3): p. 6586-606.
4. Nordin, N., S.Q. Xie, and B. Wunsche, *Assessment of movement quality in robot- assisted upper limb rehabilitation after stroke: a review.* J Neuroeng Rehabil, 2014. **11**: p. 137.
